# Maternal and Perinatal Outcomes of Pregnancy in Women With Autoimmune Disorder

**DOI:** 10.7759/cureus.16024

**Published:** 2021-06-29

**Authors:** Seema Singhal, Jyoti Meena, Sunesh Kumar, K.K Roy, Neeta Singh, Bhawani Shekhar, Anshu Yadav, Sarita Kumari, Aarthi S Jayraj

**Affiliations:** 1 Obstetrics and Gynaecology, All India Institute of Medical Sciences, New Delhi, IND

**Keywords:** pregnancy, autoimmune disorders, maternal outcome, perinatal outcome, high risk pregnancy

## Abstract

Objective

Pregnancy with an autoimmune disorder is faced with several risks for mother and fetus. The aim of the present study is to analyze the course and outcome of pregnancy in women with autoimmune disorders (AIDs).

Methods

A retrospective cohort study was conducted at a tertiary care teaching hospital. The hospital records of 153 pregnancies with autoimmune disorders and 1095 low-risk pregnant women who served as controls were reviewed. An adverse perinatal outcome was defined as the presence of any obstetric complications, including preeclampsia, eclampsia, abruption, antepartum hemorrhage (APH), prematurity, fetal growth restriction (FGR), intrauterine death (IUD), intrapartum event, mode of delivery, birth weight, neonatal intensive care unit (NICU) stay, or disease-specific neonatal complications. For all statistical tests with two-tailed probability, p<0.05 was considered statistically significant.

Results

A high incidence of adverse perinatal outcomes was observed in all women with AIDs when compared with age-matched controls. The highest incidence of adverse perinatal outcomes was observed in women with Takayasu’s arteritis. The incidence of abortions was more in women with antiphospholipid antibody syndrome (APS) and Grave’s disease (22.2% and 33.3%, respectively). The incidence of prematurity, fetal growth restriction (FGR), and low birth weight were highest in women with systemic lupus erythematosus (SLE). Pregnancy with myasthenia gravis and rheumatoid arthritis did not have any significant adverse impact on pregnancy outcomes.

Conclusion

We found a strong association between autoimmune disorders and obstetric complications. The multidisciplinary team approach and pre-pregnancy optimization of the disease improve maternal and fetal outcomes.

## Introduction

Autoimmune disorders (AIDs) are rare and six to 10 times more common in women than men [1]. They are characterized by self-reactivity of the immune system, deposition of immune complexes in target organs, and the resultant diverse clinical manifestations. The onset of the disease usually occurs in the reproductive age group and, therefore, it is not unusual for obstetricians to come across these women during their pregnancy. The pregnancy poses a challenge to both mother and fetus and is complicated in several ways, thus adding to the problems already faced [2]. Previously women with some of these disorders were advised against pregnancy but nowadays with the availability of better care, an optimum outcome can be anticipated [1]. Due to the risk of disease exacerbations and consequent adverse maternal and fetal outcomes, pregnancy is considered a high-risk condition for these patients.

AIDs have a wide spectrum, ranging from organ-specific to systemic disorders [3]. The pregnancy has a variable impact on the disease course. Pregnancy may cause an improvement of symptoms as seen in disorders mediated by T-helper (TH2) cells, such as rheumatoid arthritis and systemic sclerosis, while exacerbating or having no effect on disorders that are mediated by cell-mediated immunity such as systemic lupus erythematosus (SLE) [2]. Overall AIDs follow an unpredictable course during pregnancy with several obstetric and fetal complications [4].

Because of the rarity, there is a paucity of data on the course and outcome of pregnancy in women with AIDs. There are not enough Indian studies to assess the impact of recent advancements in the field if any. The present study was conducted to analyze the course and outcome of pregnancy in women with various autoimmune disorders.

## Materials and methods

A retrospective cohort study was conducted in the Department of Obstetrics and Gynecology, All India Institute of Medical Sciences, New Delhi. Case records of 156 pregnancies with AIDs from 2008-2017 were reviewed. The diagnosis was confirmed by a rheumatologist according to the standard disease criteria [3]. All pregnant women with AIDs who were booked and completed their treatment in our hospital were included in our study. Patients with unconfirmed or inconclusive diagnoses and those who were lost to follow-up were excluded. Demographic details, disease manifestations at the time of diagnosis, duration of disease, antibody pattern, drug intake, co-morbidities, past history, obstetric history, antibody profile, and course of disease with disease-specific events and treatment were recorded. The presence of obstetric complications, including preeclampsia, eclampsia, abruption, antepartum hemorrhage (APH), prematurity, fetal growth restriction (FGR), oligohydramnios, abnormal Doppler, intrauterine death (IUD), intrapartum event, mode of delivery, birth weight, NICU stay or disease-specific neonatal complications were recorded.

For the comparison, 1095 low-risk age-matched pregnancies without any autoimmune disorders, delivered during the study period, were taken as controls. The proportion of preterm deliveries, hypertensive disorders, gestational diabetes mellitus (GDM), and mean birth weight of controls were noted.

Statistical analysis

Data analysis was carried out using statistical software SPSS version 22.0 (IBM Corp., Armonk, NY). Descriptive statistics, such as mean and SD, were calculated for continuous variables of normally distributed data. Comparison of mean values between the groups was performed using the student t-independent test. Categorical data were expressed as frequency and percent values. The association between two categorical variables was tested using the chi-square/Fisher’s exact test. For all statistical tests with a two-tailed probability, p<0.05 was considered statistically significant.

The study was approved by the institute's ethics committee, Ref. No. IECPG-90/28.02.2018.

## Results

**Figure 1:**
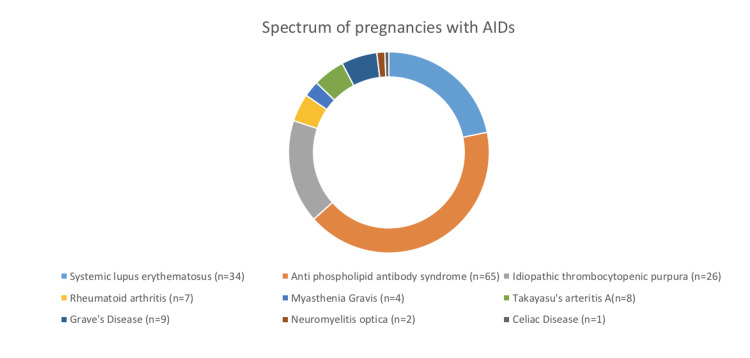
Distribution of pregnancies in women with AIDs AIDs: autoimmune disorders

The distribution of 156 pregnancies with AIDs is depicted in Figure 1. During the study period, 1095 low-risk women who delivered in the hospital were taken as controls. The controls were age-matched (mean age 26.9± 3.9 years in the case group and 27+4.1 years in the control group p=0.057). The incidence of adverse events, including preterm delivery (21.1% vs 11.2%; p=<0.001), gestational diabetes mellitus (15.9% vs 3.8% p=<0.001), and hypertension during pregnancy (8% vs 13.4% p=<0.025) was higher in women with AIDs in comparison with controls. Mean birth weight in the AIDs group was significantly lower than controls (2889.7±527.2 gm vs. 2102.3+ 612.3; p=<0.001). There was no difference in cesarean delivery rates between both groups (31% vs 25.9%; P=0.537).

There were 34 pregnancies in 21 women with SLE. The characteristics of these pregnancies are depicted in Table 1. The mean duration of illness was 5±4.2 years (0-15 years), 88.2% of women had a pre-existing disease, and only 11.8% of women were diagnosed with SLE during pregnancy. Disease status was controlled prior to conception in 64.7% (22/34) pregnancies. The course and outcome of these cases is shown in Table 2. When compared with controls, the incidence of hypertension (23.4% vs. 8%; p=0.001), prematurity (35.2% vs. 11.2%; p=0.001), and FGR (26.4% vs 1.8% (20/1095); p=0.001) was higher and the mean birth weight was lower (2067.9± 930.2 vs. 2889.7+527.2; p=0.001) in pregnant women with SLE. The NICU admission rates were as high as 31.8% in this group. The mean gestational age at the time of delivery was 35.2 ± 5.7 weeks and 50% (17/34) cases had a vaginal delivery. Twenty-two (64.7%) pregnancies resulted in live birth and five had intrauterine deaths (IUDs). Out of the five IUDs, three were unplanned pregnancies while all were premature (24 to 31 weeks) and were complicated by conditions such as chorioamnionitis, severe preeclampsia, secondary antiphospholipid syndrome (APLA), thrombocytopenia, and severe FGR (fetal weight ranging from 552-740 gm).

**Table 1:** Characteristics of pregnancies in women with systemic lupus erythematosus (SLE) and antiphospholipid antibody syndrome (APS) * antinuclear antibodies (ANA), † lupus anticoagulant (LAC), ‡ anti-cardiolipin antibodies (aCL), § hydroxychloroquine (HCQ), || recurrent pregnancy loss (RPL), ** intrauterine death (IUD), †† anti-beta 2 glycoprotein (anti-B2GP)

Characteristics	SLE (34 pregnancies in 21 women)	APS (65 pregnancies in 30 women)
	N (%) mean+ SD	N (%) mean+ SD
Age at conception (years ±SD)	28.2 + 4.2	26.09 + 3.9
Manifestations at Diagnosis N (%)	Cutaneous: 17(50)	RPL^||^: 43 (66.1)
	Articular: 15 (44.1)	Secondary APS: 11 (16.9)
	Chronic hypertension: 5 (14.7)	Prior IUD**: 5 (7.6)
	Secondary APS: 5 (14.7)	Placental insufficiency: 1 (1.5)
Autoimmune workup	ANA^*^: 34 (100)	aCL^§^: 41 (63.1)
	anti-RO/SSA-4 (11.7)	LAC^‡^: 7 (10.8)
	anti-La/SSB-6 (17.6)	Anti B2GP^††^: 11 (16.9)
	LAC^†^: 4 (11.7)	Triple positive: 5 (7.7)
	aCL^‡^: 3 (8.8)	Double positive: 2 (3.1)
Drug intake N (%)	Prednisolone: 15 (44.1)	Heparin + Ecospirin: 42 (64.6)
	HCQ^§^: 15 (44.1)	Heparin + Ecospirin+ HCQ^§^: 11 (16.9)
	Azathioprine: 5(14.7)	Ecospirin: 1 (1.6)
	Ecospirin: 12 (35.2)	No medication: 11 (16.9)
	Heparin: 8 (23.5)	

**Table 2:** Outcome of pregnancy in women with SLE, APS, and ITP * gestational diabetes mellitus (GDM), † absent end-diastolic flow/reverse end-diastolic flow (AEDF/REDF), ‡ neonatal intensive care unit (NICU), § intraventricular hemorrhage (IVH), systemic lupus erythematosus (SLE), antiphospholipid antibody syndrome (APS), idiopathic thrombocytopenic purpura (ITP)

Outcome	SLE (n=34) n(%)	APS(n=54) n(%)	ITP (n=26) n(%)
Hypertension in pregnancy	8 (23.4)	6 (11.1)	6 (23.1)
GDM^*^	1 (2.9)	nil	4 (15.4)
Prematurity	12 (35.2)	8 (14.8)	7 (26.9)
Fetal growth restriction	9 (26.4)	14 (25.9)	4 (15.3)
Oligohydramnios	3 (8.8)	8 (14.8)	Nil
AEDF/REDF^†^ in the umbilical artery	2 (5.8)	1 (1.85)	Nil
Abruption	nil	3 (5.5)	Nil
Disease-specific complication	Lupus flare: 3 (8.8%)	Thrombosis: nil	Thrombocytopenia: 21 (80.7)
Abortions	7 (20.5)	12 (22.2)	2 (7.7)
Intrauterine death	5 (14.7)	2 (3.7)	1 (3.84)
Live birth rate	22 (64.7)	51 (94.4)	23 (88.5)
Mean birth weight (g)	2067.9± 930.2	2681.4 ± 502	2382.6 ± 416
NICU^‡^ admission rates	7 (31.8)	9 (17.6)	18 (78.2)
Disease-specific neonatal complication	Neonatal heart block: 2 (5.8)	None	Thrombocytopenia: 2 (7.6); IVH^§^: nil

We observed 65 pregnancies in 30 women fulfilling the criteria for APS. Recurrent pregnancy loss was the most common presentation, seen in 66.1% (43/65) cases. Only 38 (58%) pregnancies were planned after preconceptional counseling. Eleven pregnancies were excluded from the analysis, as they did not receive any therapy. Hence, only 54 pregnancies that were treated with low-dose aspirin and prophylactic low molecular weight heparin (LMWH) starting from the first trimester were further analyzed for the perinatal outcome. Their characteristics and outcome are depicted in Table 1 and Table 2, respectively. Anticardiolipin antibodies (aCL) were the most commonly present antibodies seen in 63.1% of cases. Triple and double antibody positivity were seen in five and two pregnancies, respectively. Women with APS had a higher incidence of abortions (22.2%) and FGR (25.9%). When compared with the control group, the incidence of hypertension (11.1% vs. 8%; p=0.4), prematurity (14.8% vs. 11.1%; p=0.001), and mean birth weight (2681.4 ± 502 gm vs. 2889.7±527.2 gm p=0.004) was similar, but the risk of cesarean birth was significantly more (53% vs. 25.9%; p<0.001) in this group. The incidence of adverse outcomes was high in women who had more than one positive antibody titer. Both the cases with double-positive titers had FGR and hypertension. Similarly, out of five triple-positive pregnancies, three had abortions, the other two had FGR, and one of them delivered preterm due to severe FGR and oligohydramnios.

There were 26 pregnancies in 21 women with ITP. The mean age of women was 27.5±3.5 years. Four of them had a history of splenectomy before conception. Prior to conception, 14 pregnancies were on prednisolone therapy and two were on dapsone along with prednisolone for disease control. In 10 cases, prednisolone was started during pregnancy. The outcome of pregnancies in women with ITP is shown in Table 2. When compared with controls, the risk of hypertension (23.1% vs. 8%; p=0.006), prematurity (26.9% vs. 11.2%; p=0.001), and low birth weight (2382.6±416 gm vs. 2889.7+527.2 gm; p<0.001) was higher in women with ITP. Four pregnancies had an antepartum hemorrhage and one had an atonic postpartum hemorrhage. Intravenous immunoglobulin was administered to 10 women for correction of the acute episode of thrombocytopenia during pregnancy. Sixteen pregnancies required platelet transfusion during the antenatal period. At the time of delivery, six pregnancies had platelet counts <50,000 cells/cumm, 15 had 50,000 to 100,000 cells/cumm, and five had platelet counts >100,000 cells/cumm. The LSCS rates in these women were the same as controls (27% vs. 25.9%; p=0.91). The live birth rate was 95% with a NICU admission rate of 75%, and the duration of NICU stay ranged from three to 19 days as these neonates were monitored. Neonatal thrombocytopenia occurred in two neonates, out of which one required platelet and IVIG transfusion. None of the neonates had an intracranial hemorrhage. Overall neonatal outcome was good but one of our patients had an unexplained IUD at term.

The characteristics and outcomes of pregnancies in women with rheumatoid arthritis (n=7), myasthenia gravis (n=4), Takayasu’s arteritis (n=8), and Graves' disease (n=9) are depicted in Table 3 and Table 4. The most unfavorable outcome was observed in women with Takayasu’s arteritis, as all of them had chronic hypertension and two had eclampsia superimposed on severe preeclampsia (Table 4). A higher incidence of abortions and prematurity was seen in women with Graves' disease. Conversely, the outcome of pregnancies with rheumatoid arthritis and myasthenia gravis was favorable. None of the neonates in mothers with myasthenia gravis showed features of transient myasthenia but one of the neonates in mothers with Graves' disease had features of neonatal Grave’s, which was managed with supportive treatment. Out of the two pregnancies with neuromyelitis optica, one had an abortion and the other one had a term delivery. We came across only one pregnancy with celiac disease, and it had an uneventful course and outcome. 

**Table 3:** Characteristics of pregnancies in women with rheumatoid arthritis, myasthenia gravis, Takayasu’s arteritis, and Graves' disease * hydroxychloroquine (HCQ), † mycophenolate mofetil (MMF)

Characteristics	Rheumatoid arthritis (n=7)	Myasthenia gravis (n=4)	Takayasu’s arteritis (n=8)	Graves' disease (n=9)
Age at conception (years)(mean±SD)	27.4 ± 5.9	24 ± 2.36	22.6 ± 3.64	22.2 ± 3.4
Disease duration (years) (mean ±SD)	9.6 ± 5.7	9 ± 3	3.24 ± 4	5.6 ± 1.2
Manifestations	Swelling and tenderness of joints	Thymal carcinoma, myasthenia crisis, ptosis, facial weakness	High BP records prepregnancy, subclavian steal syndrome, bilateral carotid artery stenosis, renal artery stenosis	Exophthalmos, fatigability, weight loss, thyromegaly
Drug intake	HCQ^*^, Wysolone, Sulphasalazine	MMF^†^, Azathioprine	Alpha-methyldopa, Amlodipine, Labetalol	Propylthiouracil, Methimazole, Neomercazole

**Table 4:** Outcome of pregnancies in women with rheumatoid arthritis, myasthenia gravis, Takayasu’s arteritis, and Graves' disease * fetal growth restriction (FGR), † gestational diabetes mellitus (GDM), ^$ ^intrauterine death

Characteristics	Rheumatoid arthritis (n=7)	Myasthenia gravis (n=4)	Takayasu’s arteritis (n=8)	Graves' disease (n=9)
Abortions n(%)	1 (14.2)	1 (25)	1 (12.5)	3 (33.3)
Prematurity n(%)	1 (14.2)	nil	4 (50)	1 (11.1)
IUD^$^ n(%)	-	nil	3(37.5)	1 (11.1)
Antepartum complications n(%)	FGR^*^: 2 (28.5); Oligohydramnios: 1 (14.2)	nil	Chronic HTN: 8 (100%); Superimposed eclampsia: 2 (25); IUGR: 3 (37.5) Oligohydramnios: 1 (12.5)	GDM^†^: 1 (11.1); Oligohydramnios: 1 (11.1)
Live birth rates n(%)	7 (100)	3 (75)	4 (50)	5 (55.5)
Mean birth weight (g)	2717 ± 474	2825 ± 127	1577 ± 795	2670 ± 1112
Neonatal complications n(%)	nil	nil	Poor feeding hypoglycemia hypocalcemia: 1 (11%)	Stillbirth: 1 (11.1%); Neonatal Graves': 1 (11.1%)

The incidence of adverse outcomes in all the AIDs was higher as compared to the controls. First trimester abortions were high in all the conditions, with the highest rates in Graves' disease (33.3%), myasthenia gravis (25%), APS (22.2%), and SLE (20.6%). Takayasu’s arteritis and Graves' disease were seen to be associated with the highest incidence of adverse perinatal outcomes followed by SLE. ITP and rheumatoid arthritis had the lowest risk of adverse outcomes. Live birth rates were highest with RA and ITP followed by APS and lowest with Takayasu’s arteritis (Table 2 and Table 4).

## Discussion

Pregnancy in women with AIDs is a complex clinical situation needing multidisciplinary care and close surveillance for optimal perinatal outcomes. SLE is a multisystem disorder, and pregnancy is associated with multiple complications. In a study of 13,555 pregnancies with SLE, a significantly higher risk of preeclampsia (22.5% vs. 7.6%, p<0.001), eclampsia (0.5% vs. 0.09%; p<0.001), prematurity (20.8% vs. 8.1%; p<0.001), cesarean section (36.6% vs. 25%; p<0.001), and maternal death was observed than in non-SLE pregnant women [5]. A similar higher incidence of complications was reported by other studies as shown in Table 5 [6-10]. Studies have shown variable disease severity during pregnancy depending on the duration of disease control prior to pregnancy [11].

**Table 5:** Studies showing the comparison of outcomes in pregnant women with SLE * IUD: intrauterine death, † FGR: fetal growth restriction, ‡ LBR: live birth rate, NA: data not available

Author	Number of participants	Disease control n(%)	Lupus Flare n(%)	Preeclampsia	Preterm	IUD^*^	^†^FGR	Heart block	^‡^LBR
Agarwal et al. 1999 [6]	15	11 (73.3)	2 (13.3)	2 (13.3)	5 (33)	2 (13.3)	6 (40)	nil	10 (69.6%)
Gupta A et al. 2005 [7]	33	57.57%	9 (27.2)	3 (7.6)	2 (6.1)	nil	4 (12.1)	1 (3)	15 (45.4)
Chandran V et al. 2005 [8]	52	31 (59.6)	3 (5.7)	4 (7.8)	NA	3 (5.7)	NA	7 (3.8)	24 (46.1)
Agarwal N et al. 2011 [9]	71	71 (100)	nil	10 (28.5)	21 (29.5)	9 (13)	7 (9.8)	nil	47 (66)
Ravindran V et al. 2016 [10]	53	NA	16 (30)	1 (12.2)	9 (18.8)	2 (3.8)	9 (18.8)	nil	44 (85)
Present study 2018	34	22 (64.7)	3 (8.8)	6 (17.6)	12 (35.2)	5 (14.7)	9 (26.4)	2 (5.9)	22 (64.7)

Comparison of our results with previously published data from the same institution showed no difference in the optimization of disease activity (57.5% vs. 64.7%; p=0.5), lupus flare (27.2% vs. 8.8%; p=0.06), and live birth rate (45% vs. 64.7%; p=0.14), but a significantly higher incidence of prematurity (6.1% vs. 35.2%, p=0.006) was observed in the current study [7]. This indicates that despite improvement in multidisciplinary care, there is not much improvement in outcome and this could be due to the lack of awareness and preconception optimization of disease activity. The probability of developing neonatal lupus as a result of the passive transplacental transfer of anti-Ro and anti-LA antibodies is very low (< 1%) and the risk of congenital complete heart block was reported as 15%-30% by Izmirly et al. [12]. Conversely, we observed a lower risk of congenital complete heart block (5.8%) in our study.

Antiphospholipid antibody syndrome has the highest implications for obstetric outcomes, and standard therapy with ecospirin and low molecular weight heparin is associated with good outcomes [13]. The obstetric outcome of the present study was compared with 127 pregnant women from the Euro Phospholipid study (n=1000) cohort; a similar incidence of abortions (18.6% vs. 16.5%), FGR (25.9% vs. 26.3%), and live birth rates (78.4% vs. 72.9%) but a lower incidence of prematurity (14.8% vs. 48.2%) was observed [14]. The obstetric outcome of pregnancy with APS has not much changed over time in women who are on standard therapy. We compared the outcome of our cases with a previously published study from our center, and a similar rate of abortion (22.2% vs. 7.1%; p=0.16), FGR (25.9% vs. 9.5%; p=0.16), abruption (5.55% vs. 7.1%; p= 0.99) was seen. However, incidence of hypertension (11.1% vs. 30.9%; p=0.02) and prematurity (14.8% vs. 66.7%; p<0.001) was significantly lower in current study [13]. This could be due to improvement in antenatal care and surveillance. The most common antiphospholipid antibody in our cases was anti-cardiolipin and multiple antibody positivity was associated with worse outcomes, like other studies [15].

The overall course and outcome of pregnancy in women with ITP remain good [16-17]. In our cohort, 73% of pregnancies were on steroid therapy during their pregnancy but none of them showed any attributable malformations. Additionally, to combat an episode of severe thrombocytopenia, intravenous immunoglobulin (IVIg) was administered to 38.4% (10/26) and platelets were needed in 73.07% (19/26) pregnancies. This is in contrast with another study by Webert et al., where only 31.1% of cases needed such supportive measures [16]. None of the patients in our cohort had any severe hemorrhagic manifestations. Neonatal thrombocytopenia warranting intervention has been reported in 15% cases by Webert et al., contrary to findings of Suri et al. where none of the infants had hemorrhagic manifestations despite vaginal birth and did not receive any treatment [16-17]. Similarly, we did not observe any hemorrhagic manifestations in any of the neonates, thus confirming the safety of vaginal delivery.

Rheumatoid arthritis shows spontaneous remission in 54%-95% of pregnancies, but there is a higher risk of adverse outcomes, including small for gestational age (SGA) (3%-15%), prematurity (10%-20%), and cesarean delivery (20%-40%), than in the normal population [18-21]. Brouwer et al. reported no higher risk of miscarriage in this population [21]. We did not observe any adverse outcome in this cohort except abortion, which was as high as 41%, and this could be because of higher rates of unplanned pregnancies.

Pregnant women with Takayasu’s arteritis show high but variable rates of complications in previously published data including pregnancy loss (19%), hypertension (11%-100%), and fetal growth restriction (17%-51%) [22-25]. This variation in outcome could be because of the differential involvement of arteries by the disease in different cohorts [23]. We observed a high incidence of adverse outcomes (Table 3) in this group despite antenatal care and delivery in a tertiary care center, probably because all the pregnancies were unplanned and were not taking any treatment in the peri-conception period.

Women with Graves' disease have increased risk of adverse perinatal outcomes [26-27]. Higher miscarriages in the first trimester could be due to the transplacental passage of anti-thyroid antibodies and subsequent interference with successful implantation [1]. Aggarwal et al. reported higher odds of preterm labor (27.7% vs. 18.1%, p = 0.004), preeclampsia {OR 3.94 (95% CI- 2.47-6.29) p= 0.001}, preterm birth {1.7 (1.12-2.6), p=0.13}, FGR {2.16 (1.36-3.44), p=0.001}, cesarean delivery {1.47 (0.98-2.19); p=0.05}, and LBW (2.5 vs. 2.7 kg; p< 0.001) than controls [27]. A good control of disease before and during pregnancy reduces the risk of these adverse effects, suggesting the role of higher concentration of thyroid hormones in causing adverse outcome [26].

The overall course of myasthenia gravis remains unpredictable [28]. None of the six pregnancies in our series experienced myasthenia crisis, contrary to a study by Mitchell et al. that reported a 72.7% risk of deterioration during pregnancy [29]. Myasthenia gravis itself does not increase the risk of operative intervention [28]. Incidence of neonatal myasthenia may vary from 9%-30% [27-29]; however, we did not see any case of neonatal myasthenia in our cases.

Autoantibodies have been implicated in causing both early and late pregnancy loss. Antiphospholipid antibodies and antithyroid antibodies are specifically associated with recurrent pregnancy loss. In the current study, high rates of adverse outcomes were seen in all the disorders because of a higher proportion of uncontrolled disease and unplanned pregnancies. Another most severe complication was preeclampsia, which was present in all the variety of AIDs with the strongest association seen in APS, SLE, and Takayasu’s arteritis. The pathogenesis of this association is not clear but the role of anti-angiotensin II type I (ATI) antibodies has been implicated [4].

Study limitations

The main limitation of the present study is its retrospective nature and sample size. However, our study is novel, as it presents the outcome of a variety of autoimmune disorders. Owing to its rarity, it also provides useful evidence.

## Conclusions

There are several key issues in the management of pregnant women with AIDs and it is important for obstetricians to be aware of their course due to female predominance. We found a strong association between autoimmune disorders and obstetric complications. Pre-pregnancy optimization and multidisciplinary care is essential to get optimal maternal and perinatal outcomes.
